# RET rearrangements in post-Chernobyl papillary thyroid carcinomas with a short latency analysed by interphase FISH

**DOI:** 10.1038/sj.bjc.6603109

**Published:** 2006-04-25

**Authors:** K Unger, L Zurnadzhy, A Walch, M Mall, T Bogdanova, H Braselmann, L Hieber, N Tronko, P Hutzler, S Jeremiah, G Thomas, H Zitzelsberger

**Affiliations:** 1 Institute of Molecular Radiobiology, GSF-National Research Center for Environment and Health, Neuherberg, Germany; 2 Institute of Endocrinology and Metabolism, Academy of Medical Sciences of the Ukraine, Kiev, Ukraine; 3 Institute of Pathology, GSF-National Research Center for Environment and Health, Neuherberg, Germany; 4 South West Wales Cancer Institute, Singleton Hospital, Swansea, UK

**Keywords:** thyroid, papillary, carcinoma, FISH, chemobyl, tumour

## Abstract

Tissue samples from 13 post-Chernobyl childhood thyroid tumours that occurred within a short period of time (4–8 years) after the Chernobyl accident have been investigated by interphase FISH analysis for rearrangements of RET. In all, 77% of cases showed RET/PTC rearrangements and a distinct intratumoural genetic heterogeneity. The data were compared to findings on 32 post-Chernobyl PTCs that occurred after a longer period of time (9–12 years) after the accident. In none of the cases from either group were 100% of cells positive for RET rearrangement. In addition, the pattern of RET-positive cells was different in the two groups (short *vs* longer latency). A significant clustering of aberrant cells could be detected in the long-latency subgroup, whereas the aberrant cells were more homogeneously distributed among the short-latency tumours. The findings suggest that oligoclonal tumour development occurs in post-Chernobyl PTCs. This pattern of different clones within the tumour appears to become more discrete in cases with longer latencies, suggesting either outgrowth of individual clones or development of later subclones with time.

Rearrangements of the RET proto-oncogene, which has been localised to chromosomal band 10q11.2, represent the most frequent changes in thyroid papillary carcinomas (Santoro *et al*, 1993; Jhiang, 2000). These rearrangements termed RET/PTC result from the fusion of the RET tyrosine kinase (TK)-encoding domain to a variety of different heterologous genes that are ubiquitously expressed and are therefore able to promote the expression of the RET/PTC fusion protein. RET/PTC1 (fusion of RET with H4 gene) and RET/PTC3 (fusion of RET with RFG/ELE1 gene) are the most prevalent RET/PTC variants (Pierotti *et al*, 1996), resulting both from paracentric inversion on chromosome 10q. RET/PTC is very frequent in radiation-induced childhood papillary thyroid carcinomas developed post-Chernobyl in contaminated areas. In particular, RET/PTC3 is associated with such post-Chernobyl papillary thyroid carcinomas of solid variant and short latency (Thomas *et al*, 1999). There are several RET/PTC oncogenes that differ in the fusion partner. New types of RET/PTC have been identified in post-Chernobyl thyroid carcinomas that are generated by translocations instead of inversions like RET/PTC1 and RET/PTC3 (for a review see Jhiang, 2000).

To investigate the distribution of RET/PTC-positive cells within a particular tumour, interphase FISH analysis on paraffin-embedded tissue sections with RET-specific YAC DNA probes can be used (Unger *et al*, 2004). This type of FISH analysis has to be performed by use of laser scanning microscopy, which allows a complete scanning of cells throughout the thickness of the paraffin section. In contrast to PCR-based techniques, FISH has the possibility of detecting RET rearrangements regardless of the specific fusion partner involved and, in addition, allows examination of the presence of the rearrangement at a single-cell level.

Thyroid cancer is normally very rare in children (of the order of 0.5–1.5 per million per year), but following the Chernobyl accident it rose in those areas most heavily contaminated with radioiodine, to give a relative risk of 237 in those aged under 1 at exposure (Baverstock *et al*, 1992; Kazakov *et al*, 1992; Cardis *et al*, 1999). The incidence of thyroid cancer falls dramatically in those who were born subsequent to the accident and not exposed to radioiodine either *in utero* or in childhood (Thomas, 2003). Therefore, we can have a high degree of certainty that exposure to radioiodine was the causative agent in post-Chernobyl thyroid tumours in children, and can therefore assess the effect of latency on the pathology and molecular biology of thyroid cancer. In this study, we applied interphase FISH on a subset of post-Chernobyl tumour that became clinically apparent within a short period of time after exposure to radiation (4–8 years) and compared the data generated with that from a group of papillary cancers with a longer latency (9–12 years), in order to study the pattern of rearrangement of the RET oncogene within these two groups.

## MATERIALS AND METHODS

### Patient samples

Thirteen childhood patients (11 female, two male patients) with histologically verified thyroid tumours were studied for the presence of RET rearrangements in the tumour samples (Table 1). Appropriate informed consent was obtained from the patients or their guardians. All tumours were diagnosed as papillary carcinoma, according to the WHO classification of thyroid tumours (Hedinger *et al*, 1988). These papillary carcinomas were then further subdivided according to their dominant architecture – either papillary, follicular or solid. This categorisation has been used in other papers detailing the morphological subtypes of papillary carcinoma post-Chernobyl (Williams *et al*, 2004). Where two components were present in equal proportions, both features are given (e.g. solid – follicular papillary carcinomas are the subtype in which both solid and follicular structural components are present in the tumour in equal proportion). Patient data are summarised in Table 1. The presence of tumour cells was morphologically verified as follows: (i) slides for FISH analysis containing either tumour only or normal tissue only were obtained by removal of either areas of normal tissue or tumour tissue from the slide, resulting in tumour cell enrichment of approximately >95%; (ii) serial sections stained with haematoxylin and eosin (H and E) were used as morphological reference and (iii) after FISH signal evaluation, the very same tissue sections were stained with haematoxylin for accurate evaluation of cell content (neoplastic and non-neoplastic cells; Table 2, Figure 2). Paraffin sections (10 *μ*m) have been used for the FISH studies.

## FISH ANALYSIS

Labelling of YAC DNA probes and FISH analysis was carried as described recently (Unger *et al*, 2004). YAC probes used in this study, 313F4 and 214H10, map proximal to and include the RET locus, whereas clone 344H4 contains DNA sequences distal to RET. They were labelled either with digoxigenin-11-dUTP (344H4) or with biotin-16-dUTP (214H10, 313F4) using nick translation. Serial sections (10 *μ*m) of the tissue blocks were used for FISH analysis.

At least 100 cell nuclei per specimen (tumour and normal tissues) were scored using a confocal laser scanning microscope (Zeiss LSM 510, Zeiss, Jena, Germany). Split FISH signal (separated red and green signal) indicated a rearranged RET gene. Only cells with either two overlapping signals or one split and one overlapping signal were counted to ensure that only complete cell nuclei had been scored. Aberrant cell nuclei were identified by scoring of captured images from up to 20 viewing areas. The analysis software allowed a stepwise scoring every 0.5 *μ*m to ensure that signals in different layers of the sections were evaluated accurately.

Positive and negative controls were used as described previously (Unger *et al*, 2004). As a positive control, the TPC-1 cell line carrying a RET/PTC1 rearrangement was fixed in formalin and embedded in paraffin, sectioned and treated as per the FISH protocol used on the paraffin sections from tumours. The paraffin-embedded TPC-1 cell line showed rearrangement-positive FISH signals in 97% of nuclei counted (100 out of 103 cells scored). This was shown to be reliable when repeated in different experiments. As a negative control, sections of normal colon mucosa as well as four RET/PTC-negative cell lines (S414N, a normal thyroid cell line, RPE, a retina pigment epithelial cell line, the SHSY cell line and the triploid NPA cell line), embedded in paraffin, and hybridised with FISH probes for RET using the same protocol as was used for the thyroid sections. These results were consistently negative.

## STATISTICAL ANALYSIS

To determine the cutoff level that represents a significantly elevated frequency of *RET*-rearranged cells compared to the baseline frequency in normal thyroid, mean value (x̄) and standard deviation (s.d.) of RET-rearranged cells in sections from normal thyroid were calculated. The resulting mean value was 2.4±2.1. For calculation of the cutoff level, a value of 3x̄ equivalent to 7.1% of aberrant cells was accepted.

**Figure 1:**
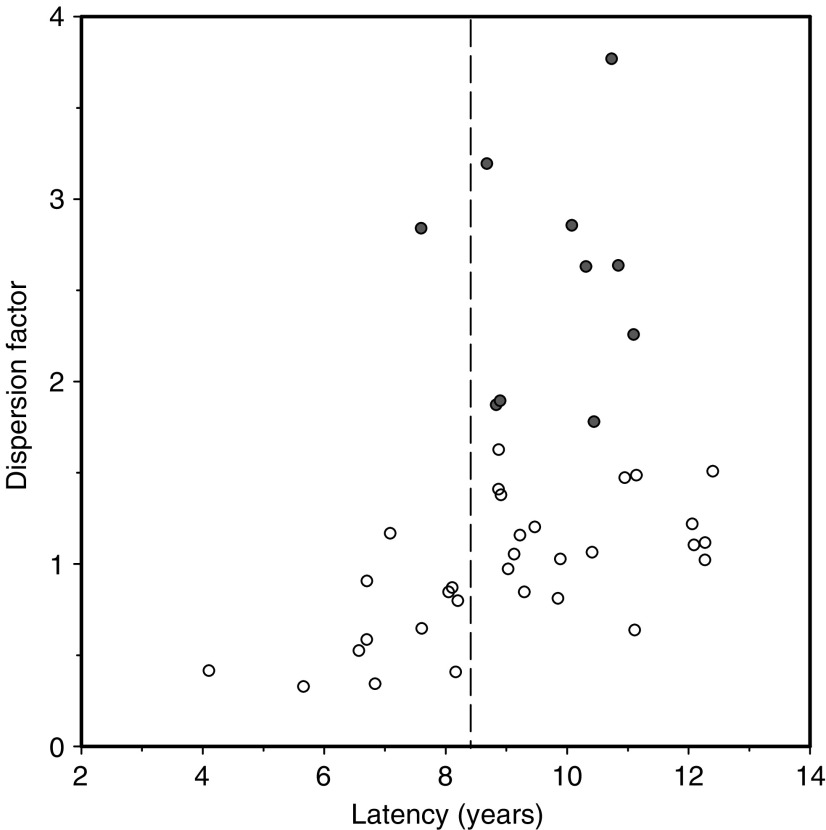
Scatter plot showing the dispersion parameter for RET/PTC-rearranged cells within tumours against latency for each tumour patient. Filled circles represent significantly elevated dispersion factors indicating cases with a nonhomogeneous distribution of RET/PTC-rearranged cells within tumours. It is clearly visible from these diagrams that RET/PTC clustering occurs only in tumours with a latency of >8 years after the accident. These results are the basis for the discrimination in cases of short (4–8 years) and long (9–12 years) latency.

The binominal homogeneity test was applied for an analysis on the distribution of aberrant FISH signals within different viewing areas of a particular case. A dispersion factor was calculated and *P*-values were derived which indicated whether the distribution of aberrant FISH signals was significantly different from a homogeneous distribution. If this was the case, a clustering of aberrant cells was postulated. A scatter plot of dispersion factors against latency is shown in Figure 1, demonstrating that a clustering of RET/PTC-rearranged tumour cells (significantly elevated dispersion factors, red dots) occurs only in cases with a latency >8. This finding led us to discriminate the tumour cohort in two groups, one with a short latency (4–8 years after exposure) and another with a long latency (9–12 years after exposure).

## RESULTS

**Figure 2:**
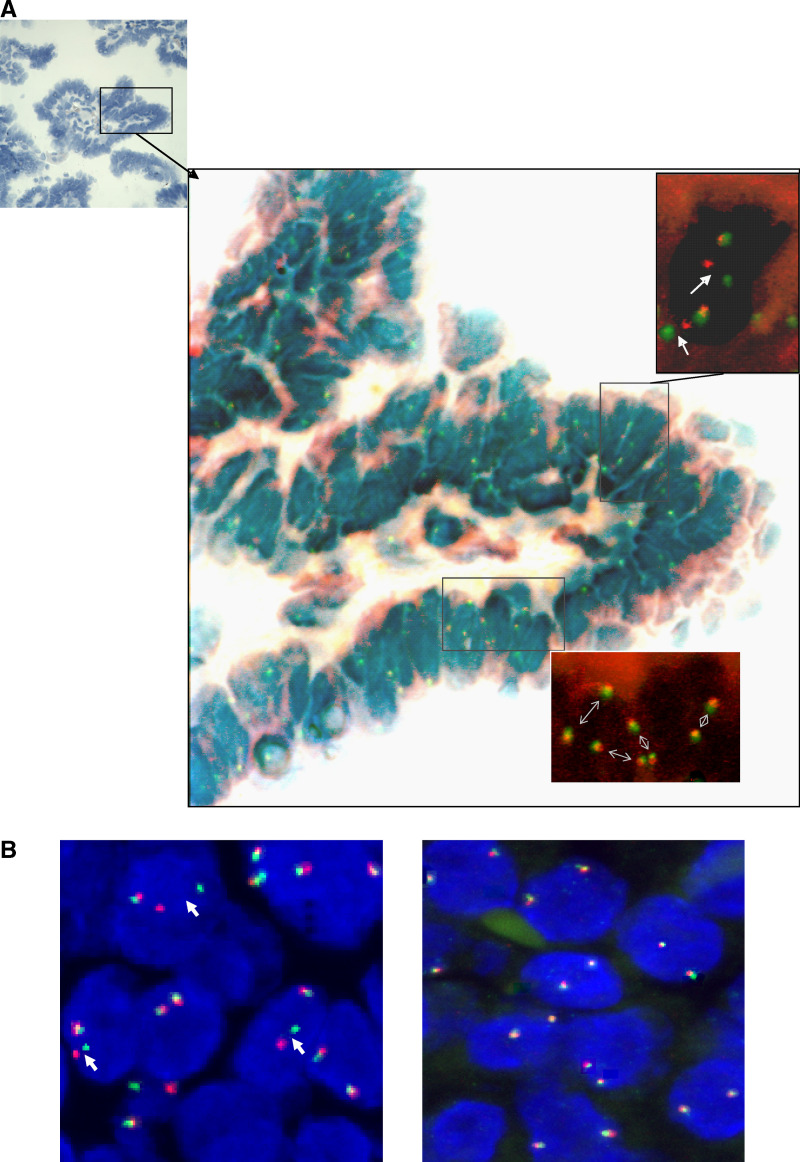
FISH analysis with RET-specific DNA probes on paraffin sections using confocal LSM. (**A**) Hybridised section of case III stained simultaneously with hematoxylin to evaluate histologic features of the FISH-scored area. A papillary structure consisting of epithelial tumour cells in the vast majority. Examples of FISH-scored areas containing RET/PTC-rearranged and nonrearranged tumour cells are indicated. (**B**) Examples from two different tumour areas of the same case are demonstrated. One viewing area shows split FISH signals (arrows, left), and another viewing area shows only normal cells exhibiting overlapping FISH signals. All images are superimposed from approximately 10 different slices throughout the thickness of the tissue section.

We have investigated 13 post-Chernobyl PTCs by FISH interphase analysis for the presence of RET rearrangements. We used a combination of YAC DNA probes that were labelled in two different colours and hybridised paraffin-embedded tissue sections. A split FISH signal in red and green within a cell nuclei in addition to an overlapping signal (mixed colour, yellow) indicates a rearranged RET gene, whereas normal cells show two overlapping signals (Figure 2). To provide enriched tumour cells on the hybridised slides for subsequent LSM scoring, the paraffin sections had been dissected to provide either areas of tumour only (>95%) or normal tissue only before use (Figure 2). FISH allows to examine RET rearrangements at a single-cell level and provides the advantage that RET rearrangements can be detected regardless of the specific fusion partner gene involved.

**Figure 3:**
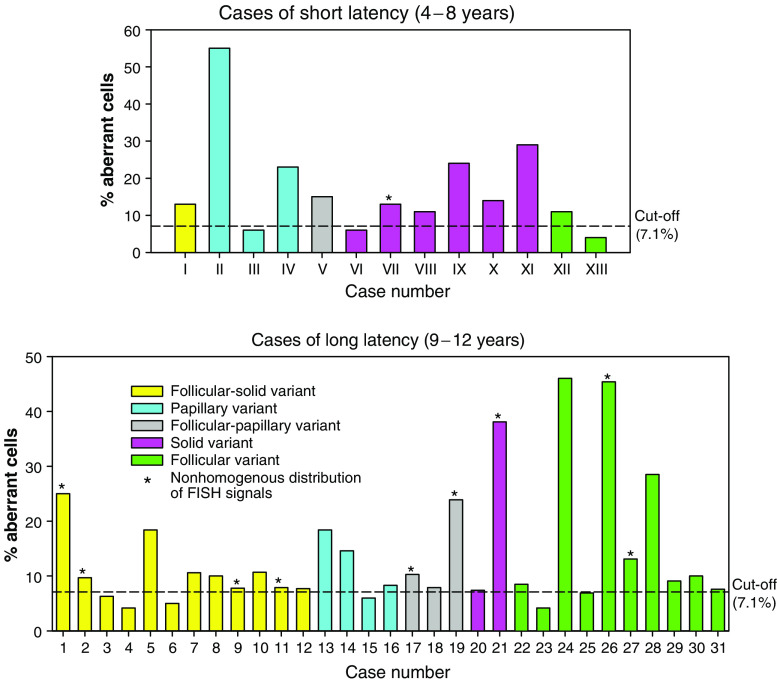
Frequency of RET/PTC-positive cells detected by interphase FISH analysis in post-Chernobyl papillary carcinomas of short (upper graph) and long (lower graph; Unger *et al*, 2004) latency. Histological subtypes are classified in different colours. Cases exhibiting a statistically significant clustering of RET/PTC-positive cells are indicated by asterisks.

**Figure 4:**
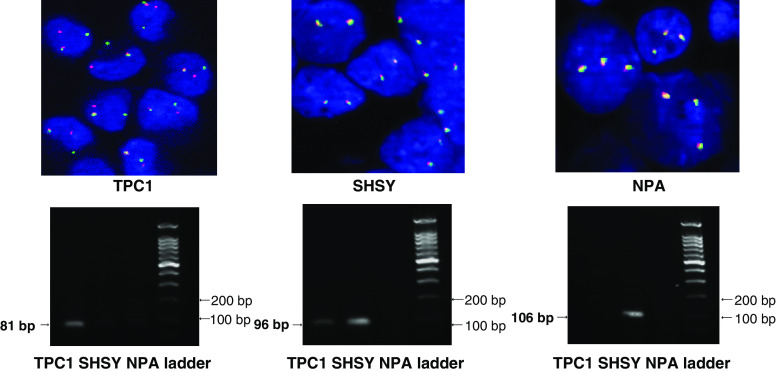
Analysis of RET/PTC rearrangements on control cell lines. (**A**) interphase FISH with YAC DNA probes on TPC-1 (left), SHSY (middle) and NPA (right) cell lines. All interphase cell nuclei of TPC-1 exhibit a split FISH signal and an overlapping FISH signal suggesting RET/PTC rearrangement, whereas exclusively overlapping FISH signals are visible in SHSY and NPA cells (**B**) RT–PCR for RET/PTC1 (left), TK domain (middle) and extracellular domain (right). RET/PTC1 rearrangement is only detectable in TPC-1 cells, whereas expression of the RET TK domain is observed in both TPC-1 and SHSY cells. Extracellular domain expression is only observed in SHSY cells, which express the full-length c-RET transcript. TPC-1 is positive for RET/PTC1 (positive control), SHSY shows expression of full-length RET without rearrangement (negative control) and the triploid NPA cell line exhibits neither RET expression nor RET rearrangement by FISH (negative control).

The frequency of aberrant cells (RET/PTC) is indicated in Table 2 and Figure 3 for 13 post-Chernobyl PTCs. Ten of 13 cases (77%) have been diagnosed as RET-rearranged taking a 7.1% threshold for significant distinction from background frequencies into account. The highest observed frequency after interphase FISH analysis was 55% (case III). None of the post-Chernobyl tumours showed a uniformly positive rearrangement signal in all epithelial cells. To demonstrate the reliability of the interphase FISH approach used, we performed a series of control experiments on formalin-fixed, paraffin-embedded TPC-1, SHSY and NPA cells (Figure 4). The positive control (TPC-1 cell line) produced a positive rearrangement signal in 97% of nuclei counted in three different experiments. Split FISH signals were absent in all negative controls (cell lines without RET/PTC rearrangement or with expression of the wild-type RET). The interphase FISH results were consistent with RT–PCR results from the same cell lines with identical passage numbers. A correlation of the frequency of rearranged cells with histological subtypes of PTC could not be observed. A statistically significant clustering of rearranged cells became obvious for case VIII, which showed a deviation from homogeneous distribution of split signals among all viewing areas (distribution homogeneity test). Thus, as demonstrated in Table 2 and Figure 3, only one case out of 13 (8%) from the short-latency group shows a nonhomogeneous distribution of FISH signals, indicating clustering of cells harbouring a RET rearrangement. In the longer latency group, nine out of 32 cases (28%) exhibited this phenomenon (Figure 3; Unger *et al*, 2004).

## DISCUSSION

RET rearrangements have been investigated by interphase FISH analysis in 13 post-Chernobyl PTCs. FISH analysis have been performed by use of laser scanning microscopy unlike a previously reported study in sporadic PTCs that used conventional fluorescence microscopy (Cinti *et al*, 2000).

The post-Chernobyl PTCs investigated in this study became clinically apparent within 4–8 years following exposure to radioiodine in fallout. We have already published a similar investigation on 32 cases with a longer-latency of 9–12 years after irradiation (Unger *et al*, 2004). Comparison of data from both studies shows similar frequencies of RET-rearranged cases (72% in the long latency group *vs* 77% in the shorter latency group). Thus, there is no significant difference in the frequency of cases that harbour a RET rearrangement between tumours of differing latencies after irradiation. However, if the distribution of cells harbouring a RET rearrangement within tumours are considered, remarkable differences between tumours of differing latencies become apparent. These findings indicate that short-latency cases show an interspersal of RET rearrangement-positive epithelial cells with those that do not harbour a RET rearrangement, whereas the longer latency group show a pattern that could be associated with the development of subclonal outgrowth, or consistent with RET rearrangement occurring as a second event in subclones of a pre-existing lesion. This observed heterogeneity is unlikely to be an artefact because only cells with either two overlapping (indicating no rearrangement involving chromosome 10) or one overlapping and a split signal (indicating the presence of a rearrangement of chromosome 10) were scored. Cells in which there was only one signal were excluded from analysis to avoid any artefacts owing to section preparation. In an earlier study, it has been demonstrated that in 35% of normal human thyroid cells at least one pair of RET and H4 signals were juxtaposed detected by FISH analysis (Nikiforova *et al*, 2000). Thus, a significant number of tumour cells with RET/PTC rearrangement might be misclassified, which is dependent on the interphase arrangement of chromosome 10 in the tumour nuclei and the linear distance between RET and the partner gene. To avoid this problem, we have used in our study a different FISH approach showing overlapping FISH signals in normal nuclei and split signals only when the RET gene is disturbed. This approach avoids confusion caused by the interphase arrangements of chromosome 10 as described by Nikiforova *et al* (2000). We also have performed a series of control studies that argue against artefacts (caused by the nuclear arrangement of chromosome 10 or by formalin fixation). As demonstrated in Figure 4, RT–PCR and interphase FISH results are in good agreement in control cell lines.

The reported genetic heterogeneity in our study is a well-known phenomenon in solid tumours and even in thyroid lesions (Ferrer-Roca *et al*, 1998; Fusco *et al*, 2002) and indicates that clonal evolution in such tumours is a complex process. These findings on post-Chernobyl PTCs developed with short and long latency after irradiation suggest that multiple clones develop from follicular cells to form papillary cancer. This interpretation is supported by other studies on papillary cancers (Sugg *et al*, 1998; Tallini *et al*, 1998; Fusco *et al*, 2002) and the recent demonstration that the histology of post-Chernobyl PTCs is inhomogeneous and is changing with time after the accident (Williams *et al*, 2004). This is borne out in this study, as the proportion of tumours of a solid-follicular morphology is increased in those of longer latency, and those of a purely solid morphology is decreased (Figure 3). Interestingly, a similar pattern of subclonal involvement of the RET oncogene has been suggested by a study in medullary carcinoma (Eng *et al*, 1998). These studies, in humans, would appear to conflict with studies in animals using the X-chromosome as a marker of clonality (Thomas *et al*, 1989). However, one possible explanation to reconcile these differences is that thyroid carcinomas do indeed derive from a single cell, but that intratumoural heterogeneity among subclones of the initial clone develops early in tumour neogenesis. Proof of this hypothesis would require identification of the initiating event in thyroid carcinogenesis. The fundamental mutation should be present in all epithelial cells within the tumour, with genetic alterations that were acquired subsequently present in only a proportion of the cells within the tumour. This study, together with our previous study, would indicate that RET rearrangement is not the initiating event in papillary carcinomas associated with radiation exposure following the Chernobyl accident.

In conclusion, the comparison of the *in situ* pattern of RET rearrangement in groups of tumours with different latencies postexposure to radioiodine in fallout from the Chernobyl accident shows distinct differences and supports either a polyclonal development for papillary carcinoma or early development of subclonal diversity.

## Figures and Tables

**Table 1:** Information on 13 patients on age at diagnosis, exposure and the latent period

**Case**	**Age at exposure (years)**	**Age at operation (years)**	**Latency (years)**
Mean	6.41	13.69	7.34
Median	6.84	14.09	7.61
Range	4.16–7.95	12.27–16.00	5.67–8.21

**Table 2:** FISH analysis of RET rearrangements in 14 post-Chernobyl PTCs with a short latency

			**Tumour tissue**			
**Case**	**Latency in years/gender^a^**	**Histological variant**	**Aberrant cells (%)**	**Normal cells (%)**	**Nonhomogeneous distribution of FISH signals^b^**	**Tumour cell content^c^ (%)**
I	6.58/F	Follicular/solid	13	87	—	>95
II	8.21/F	Papillary	55	45	—	>95
III	8.06/F	Papillary	6	94	—	>95
IV	5.67/F	Papillary	23	77	—	>95
V	8.18/F	Papillary/follicular	15	85	—	>95^d^
VI	8.12/F	Papillary	6	94	—	>95
VII	7.61/F	Solid	13	87	Yes	>95
VIII	7.10/F	Solid	11	89	—	>95^d^
IX	7.62/M	Solid	24	76	—	>95
X	8.0/F	Solid	14	86	—	>95^d^
XI	6.85/F	Solid	29	71	—	>95^d^
XII	6.72/M	Follicular	11	89	—	>95
XIII	6.71/F	Follicular	4	96	—	>95^d^

a Years/F=female; FISH=fluorescent *in situ* hybridisation; M=male.

b Binominal homogeneity test to calculate a dispersion factor. *P*<0.05 indicates a nonhomogeneous distribution of aberrant cells between all viewing areas scored.

c The percentage of tumour cells was evaluated from microdissected tissue sections after FISH analysis and after staining with haematoxylin (see also Figure 1).

d Sporadic entrapped non-neoplastic follicles were present (<1–2%).
